# Effects of Extremely Low Frequency Magnetic Field Exposure (50 Hz, 200 µT) on Cell Viability, DNA Damage and Micronucleus Formation of Human Skin Cells

**DOI:** 10.1002/bem.70046

**Published:** 2026-02-05

**Authors:** Vivian Meyer, Karen Drees, Isabel Alexandra Gronau, Alexander Lerchl

**Affiliations:** ^1^ School of Science Constructor University Bremen Germany; ^2^ Department of Biology and Environmental Sciences Carl von Ossietzky Universität Oldenburg Oldenburg Germany

**Keywords:** DNA, ELF‐MF, human, in vitro, keratinocytes, skin

## Abstract

Both everyday electrical devices and high voltage transmission lines produce electric and magnetic fields with a frequency of 50 Hz in Europe and most other countries. Although several studies have already been investigating the effects of extremely low frequency magnetic field (ELF‐MF) exposure on biological material, this topic is still debated. High‐quality research is still needed to keep the available data up to date and to decrease the low‐quality study design bias. We investigated the effects of 50 Hz magnetic fields with an intensity of 200 µT (rms) on HaCaT cells, an immortal keratinocyte cell line derived from adult human skin cells. The exposure system allowed standard in vitro cultivation and blinded experimental design with simultaneous exposure and sham‐exposure for 2 or 24 h. The biological endpoints were measured using the WST‐1 assay (cell viability), the alkaline comet assay (DNA integrity), and the micronucleus test (chromosomal distribution). The results show no significant effects of the parameters mentioned. Our data support the assessment that 50 Hz ELF‐MF up to 200 µT do not cause health effects. This study contributes valuable knowledge to the existing pool of evidence for the effects of ELF‐MF on human cells.

## Summary

- Human keratinocyte cells (HaCaT) were exposed to 50 Hz, 200 µT magnetic fields to assess potential genotoxic effects.
- No significant effects were found on cell viability, DNA damage (comet assay), or chromosomal maldistribution (micronucleus test).
- The results support the conclusion that exposure to 50 Hz ELF magnetic fields up to 200 µT does not pose a health risk.

## 1 Introduction

Today, increasing numbers of electrical devices and applications lead to high energy demands. Power networks will be expanded to transport renewable energy from offshore and coastal regions (wind turbines) and desert regions (photovoltaic systems) to end users. Both everyday electrical devices and high voltage transmission lines produce electric and magnetic fields with a frequency of 50 Hz in Europe and most other countries. This raises the question of whether exposure to these extremely low frequency (ELF) fields has adverse health consequences. The fact that, in addition to the frequency of 50 Hz, harmonics (integer multiples of the fundamental frequency) also occur for physical and technical reasons is not widely known (Fiocchi et al. 2015; Isokorpi et al. 2000; Kurokawa et al. 2003; Paniagua et al. 2004). These are caused, for example, by transformers, switching power supplies or dimmers. Due to their non‐linear current consumption, these devices generate harmonics, usually with a dominant proportion of the 3rd, 5th, and 7th harmonics (i.e., 150 Hz, 250 Hz, 350 Hz).

Based on pooled analyses of epidemiological research that reported an association between exposure to low‐level magnetic fields and childhood leukemia, the International Agency for Research on Cancer (IARC) classified ELF magnetic fields (ELF‐MF) as possibly carcinogenic to humans (IARC 2002). This was empowered by studies that found effects on various biological endpoints like DNA strand breaks and an increased number of micronuclei (e.g., Ivancsits et al. 2002; Simkó, Kriehuber, and Lange 1998). On the contrary, several other studies could not confirm these findings but stated that ELF‐MF do not alter the same biological endpoints (e.g., Scarfi et al. 1999; Stronati et al. 2004), which led to inconclusive reviews (e.g., Vijayalaxmi and Obe 2005). In an elaborate study design, four controversial in vitro studies were replicated in a regional electromagnetic field exposure facility under carefully controlled experimental conditions (Boorman et al. 2000). Interestingly, none of the formerly reported effects on gene expression, intracellular calcium concentration, cell colony growth, and ornithine decarboxylase activity could be confirmed. Considering these contradictory study results, the WHO convened a Task Group of scientific experts to assess any risks to health that might exist from exposure to ELF fields (> 0 to 100 kHz), reviewing evidence for several health effects, and updating the evidence regarding cancer. Following a standardized health risk assessment process, the Task Group concluded that there are no substantive health issues related to ELF fields at levels generally encountered by members of the public (Repacholi 2012; Van Deventer 2007).

To date, there are still studies that find possible effects of ELF‐MF on health. Ersoy et al. (2022) postulated that ELF‐MF exposure during embryonic development and adolescence of Sprague–Dawley rats can cause apoptosis and structural changes in the testis. There is a recent review that states that the majority of studies on ELF‐MF found genotoxic effects (Lai 2021).

Consequently, these controversial discussions call for further high‐quality research to investigate possible biological effects of ELF‐MF to keep the available data up to date and to decrease the low‐quality study design bias. In this project, the influence of low‐frequency fields on human skin cells was investigated. A special focus was on cell viability and the occurrence of genomic damage (DNA strand breaks and chromosomal maldistribution).

## 2 Materials and Methods

### 2.1 Cell Culture

One batch of HaCaT cells (CLS Cell Lines Service, Eppelheim, Germany) were obtained in passage 31 (Boukamp et al. 1988), cultivated, and pooled in passage 33 before freezing several aliquots. Cells were cultured in DMEM, high glucose, GlutaMAX (Thermo Fisher Scientific, Waltham, USA) supplemented with 10% FBS (Gibco, Waltham, USA) and 1% Penicillin Streptomycin (Thermo Fisher Scientific, Waltham, USA) in T25 Tissue Culture Flasks (Corning, New York, USA) in an incubator with 37°C, 5% CO_2_ and saturated humidity. Medium was changed twice a week, and cells were subcultivated once a week. Subcultivation was performed by washing the cells once with 1x phosphate‐buffered saline (PBS; Gibco, Waltham, USA) and incubating them with 2 mL TrypLE Express (Gibco, Waltham, USA) for 7 min at 37°C. TrypLE procedure was stopped by adding 4 mL medium. Cells were centrifuged for 5 min at 300*g*, resuspended in medium, and split 1:15 before seeding. For the experiments, cells were used in passages 34–37.

### 2.2 Exposure System and Experimental Design

**Figure 1:**
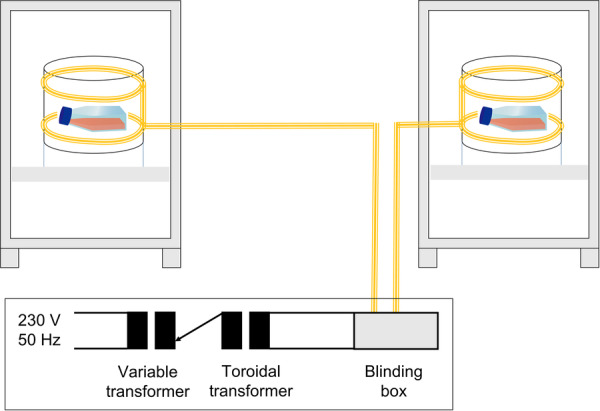
Schematic drawing of the exposure system showing the two incubators with the two Helmholtz coils and the cell culture flasks as well as the wiring diagram. The upper part is not to scale.

**Figure 2:**
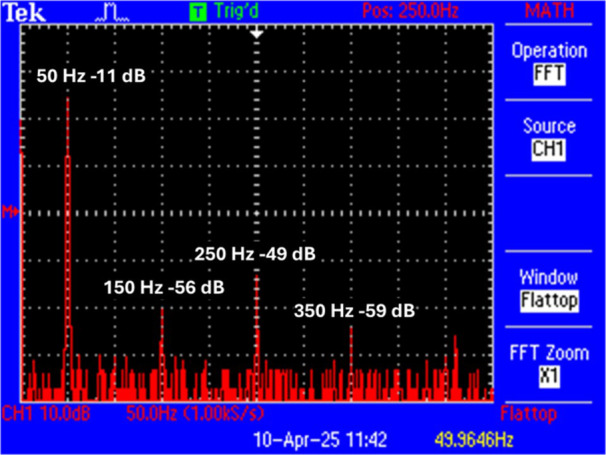
Screenshot of the FFT analysis of the ELF‐MF showing the presence of the harmonics. Overall, the proportion of harmonics amounted to approx. 1.5% of the total magnetic field.

The exposure system was designed for standard in vitro incubation during exposure. The system consisted of two similar incubators (MCO‐80IC‐PE, PHCbi, Tokyo, Japan) in which Helmholtz coils were integrated (Figure 1). They consisted of round plastic tubes as holders with a diameter of 20 cm and twisted double copper strands of four turns each at a vertical distance of 10 cm. The electric fields from the coils were eliminated by aluminum foil, which covered the plastic holders. The DC geomagnetic fields inside the incubators at the locations of the cell dishes were almost identical (57 and 56 µT, respectively). There was no measurable leakage of magnetic fields from one incubator to the other incubator. At a distance of 2.5 m between the dishes, the stray field from the verum cells was less than 0.01%. The power supply was provided by connecting a variable transformer and a toroidal transformer in series. At 230 V mains voltage, an output voltage of 18.1 V (rms) was set on the variable transformer (type 5315.0, ics Schneider Messtechnik, Bergfelde, Germany), which was transformed to 2.2 V (rms) by the toroidal transformer (R 1000‐S, Transtec, Hilden, Germany). At 5.6 A (rms), the coils generated a magnetic field of 200 µT (rms), which was measured by field probes (FLC 100, Stefan Mayer Instruments, Dinslaken, Germany). The current was measured using a current clamp (TA189, pico Technology, Cambridgeshire, UK) and an oscilloscope (TPS2014, Tektronix, Beaverton, OR). The harmonics were measured using FFT analysis on the oscilloscope (Figure 2). It turned out that 3rd, 5th, and 7th harmonics are clearly present at 150, 250, and 350 Hz, respectively. In total, they accounted for 1.5% of the total field intensity. Exposure and sham‐exposure were performed simultaneously under blinded conditions and with randomized assignment to the exposure systems. Exposure conditions were balanced over the four replicates (i.e., twice sham and twice verum in each exposure system). The sham‐exposure was realized by neutralizing the magnetic field through antiparallel winding. The residual magnetic field in the sham‐exposure coils was less than 0.2 µT. The AC (50 Hz) background magnetic fields in the incubators were measured at steady state, that is, after the desired temperatures were reached, and were caused by the ventilators; they were < 0.30 µT. The cells were exposed to a magnetic field of 50 Hz and 200 µT (rms) or sham‐exposed for 2 or 24 h. We selected an exposure level of 200 µT because this value of magnetic flux density is the reference level for general public exposure for 50 Hz magnetic fields recommended by the International Commission for Non‐Ionizing Radiation Protection (ICNIRP) in their guidelines published in 2010, and it lies clearly above typical environmental background exposure. Using 200 µT allowed us to (i) ensure a robust signal‐to‐noise ratio for controlled laboratory conditions, and (ii) remain within a range that is technically feasible and biologically relevant. Four replicates per treatment were processed. The data were analyzed for normal distribution by the Shapiro–Wilk test. Since all data were normally distributed, Student's *t*‐test (two‐tailed, unpaired) was chosen to check for statistical differences.

### 2.3 Cell Viability Assay

The cell viability assay was performed using Cell Proliferation Reagent WST‐1 (Roche, Basel, Switzerland) according to the manufacturer's manual. First, different cell densities from 100 to 50,000 cells and WST‐1 reagent incubation times from 0.5 to 4 h were tested. A seeding density of 3000 cells per well was found to be in the linear range of the standard curve of absorption measurements after 4 h (data not shown). Therefore, for the experiments, 3000 cells in 100 µL medium per well were seeded in five wells of two microtiter plates for simultaneous exposure and sham exposure (TC‐Plate 96 Well, Sarstedt, Nümbrecht, Germany), and neighboring five wells were each filled with 100 µL cell culture medium only for blank. For each set of replicate experiments, we thawed one aliquot of cells and used them for each of the four replicate experiments in different passages. Cells were grown for 1 (24 h experiments) or 2 days (2 h experiments) and then placed in the exposure system for the respective time interval. Positive controls were grown for 2 days, treated with freshly prepared 10 mM H_2_O_2_ in cell culture medium for 20 min at room temperature (RT), washed with medium, and fresh medium was added. Sham positive controls were treated simultaneously and similarly, but with cell culture medium only for 20 min at RT. After exposure, sham‐exposure, or treatment, 10 µL WST‐1 reagent was added to each of the 10 wells per plate and incubated for 4 h at 37°C. Absorption measurements of the cell medium in each microtiter plate well were performed after shaking the plates for 60 s using a microplate reader (Tecan Sunrise, Männedorf, Switzerland). The measured cell medium originated from all cells in that particular well. In each experiment, the absorption measurement of each sample well was reduced by the measured absorption of its neighboring blank well. Relative absorption was calculated by setting the mean measured absorption of each sham replicate to 100% and calculating the percentage values for each of the five measurements per exposure replicate, respectively. Student's *t*‐test was used to test for significant differences between the measured absorption of exposed and sham‐exposed samples in each of the four replicate experiments per exposure time group.

### 2.4 Comet Assay

The comet assay was performed using the CometAssay Single Cell Gel Electrophoresis Assay (R&D Systems, Minneapolis, USA) according to the manufacturer's protocol. For each set of replicate experiments, we thawed one aliquot of cells and used them for each of the four replicate experiments in different passages. Briefly, cells were seeded 5–6 days before exposure in T25 Tissue Culture Flasks (Corning, New York, USA) with a change of cell culture medium at Day 3. Cells were then placed in the exposure system for 24 or 2 h, respectively. After exposure or sham‐exposure, all cells of each flask were detached following the above‐described protocol for subcultivation, but with TrypLE incubation for only 3 min and centrifugation for only 3 min to reduce cell damage. A cell suspension of 1000 cells was centrifuged again at 300*g* for 3 min and resuspended in 1 mL PBS (Gibco, Waltham, USA). Positive controls were included in each experiment. Positive control cells were seeded and detached as described above; the cell pellet of 1000 cells was resuspended in 1 mL freshly prepared 100 µM H_2_O_2_ in PBS and incubated for 20 min at RT. Sham positive controls were treated simultaneously and similarly, but with PBS only for 20 min at RT. Of each cell suspension, 5 µL were transferred to a prewarmed tube, resuspended with 50 µL Low Melting Agarose (R&D Systems, Minneapolis, USA), and transferred to one well of a prewarmed CometSlide (R&D Systems, Minneapolis, USA). After 30 min of solidification at 4°C, the slides were incubated overnight in Lysis Solution (R&D Systems, Minneapolis, USA) at 4°C. Slides were washed and then incubated for 1 h in alkaline solution (300 mM NaOH) and 1 mM Ethylenediaminetetraacetic acid (EDTA) in demineralized water (R&D Systems, Minneapolis, USA) at 4°C. Finally, electrophoresis was performed in alkaline solution with 1 V/cm and 250 mA for 30 min at 4°C. Slides were then incubated twice in demineralized water for 5 min, in 70% ethanol for 5 min, and then dried at 37°C. To stain the nucleus and DNA fragments, 1 µL 10.000x SYBR Gold in DMSO (Invitrogen, Waltham, USA) was diluted in 30 mL demineralized water for a 30 min incubation of the slides in the dark.

Microscope images were taken with an AxioSkop2 Plus microscope with AxioCam and the associated software AxioVision 7.4 (Carl Zeiss, Oberkochen, Germany) at a magnification of 25x with filter set 09 for SYBR Gold (Figure 4A,B). The images were processed with OpenComet v1.3.1 (Gyori et al. 2014) in ImageJ (2.0.0‐rc‐69/1.52p/Java1.8.0_172 (64 bit); National Institutes of Health, USA; Figure 4C,D), and the relative DNA in the tail as a percentage of DNA in the whole comet was compared between exposed and sham‐exposed samples with Student's *t*‐test. Positive control data of the 2 h experiments were compared to the respective 2 h sham samples.

### 2.5 Micronucleus Test

For the micronucleus test, we used cells of two aliquots per set of experiments; more precisely, we used the cells of one aliquot for two experimental replicates in different passages. In each experiment, 400,000 cells were seeded in detachable slide flasks or 4‐well on glass (Sarstedt, Nümbrecht, Germany), grown for 1 (24 h experiments) or 2 days (2 h experiments), and then placed in the exposure system for the respective time interval. Positive control samples were grown for 2 days and then treated with 1.5 µM mitomycin C (Carl Roth, Karlsruhe, Germany) in fresh cell culture medium for 4 h at 37°C (according to Hintzsche et al. 2012). After exposure/treatment or sham‐exposure/‐treatment, the medium was changed, the cells were washed once with fresh medium, and 3 µg/mL cytochalasin B (Serva Electrophoresis, Heidelberg, Germany) in medium was added. After incubation for 1 day at 37°C, flasks or wells were detached from slides, cells were fixed in ice‐cold methanol at −20°C for at least 1 h, and slides were air‐dried for 20 min. Slides were washed with PBS (Gibco, Waltham, USA) and blocked with 1% albumin from bovine serum (Sigma, Burlington, USA) in PBS for 15 min, both at RT. First antibody (human anti‐centromere/kinetochore; Antibodies, Davis, USA) was diluted 1:100 in PBS, transferred to the slides that were encircled with a Super PAP Pen (Daido Sangyo, Tokyo, Japan), and slides were incubated for 1 h at 37°C. After three washing steps with PBS at RT, secondary antibody (fluorescein‐labeled chicken anti‐human IgG; Aves Labs, Davis, USA) diluted in PBS was transferred to the slides and incubated for 30 min at 37°C. Another three washing steps with PBS at RT completed the protocol, and slides were embedded with mounting medium ROTI Mount FluorCare DAPI (Carl Roth, Karlsruhe, Germany).

Microscopic analysis was performed with an AxioSkop2 Plus microscope (Carl Zeiss, Oberkochen, Germany) at a magnification of 63x (oil immersion) with filter set 49 for DAPI and 09 for FITC. Per replicate, 1000 binucleated cells (BN) of the whole cell monolayer were analyzed. If micronuclei appeared, BN with CREST‐positive (MN+; Figure 5A) and CREST‐negative micronuclei (MN‐; Figure 5B) were counted. The criteria for micronuclei were the following: staining of the main nucleus and micronucleus is similar; main nucleus and micronucleus do not overlap; size of micronucleus does not exceed one third of the main nucleus (Fenech et al. 2003). Differences between exposed and sham‐exposed samples were tested for significance with Student's *t*‐test.

## 3 Results

### 3.1 Cell Viability

**Figure 3:**
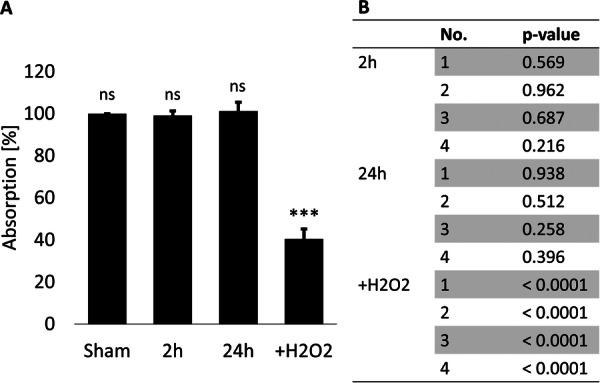
Results of absorption measurements for cell viability assessment. (A) Diagram showing the mean relative absorption per exposure time group and positive control (+H_2_O_2_); bars: standard deviation. (B) Table showing the results of Student's *t*‐test (*n* = 5 for each replicate) when testing for differences between exposure or treatment and corresponding sham control for each of the four technical replicates per group. No.: replicate number.

The mean relative absorption after addition of WST‐1 reagent to the cells was similar for sham exposure samples and both exposure time groups, whereas the samples of the positive control showed an absorption of less than 50% of the sham treatment samples (Figure 3A). The measured absorption was not different between exposure and sham exposure after 2 h or 24 h exposure in any of the replicates, whereas highly significant differences were found between the measured absorption of positive control treatment and sham treatment for each of the technical replicates (*n* = 4; Figure 3B).

### 3.2 DNA Damage

**Figure 4:**
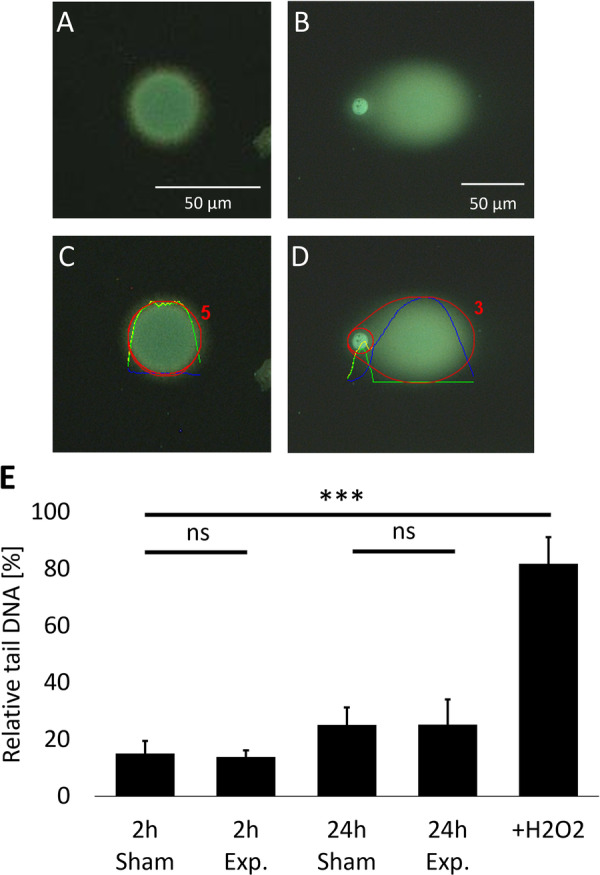
Results of comet assay for DNA strand break measurement. (A–D) Images of cell nuclei after electrophoresis stained with SYBR Gold and analyzed with OpenComet software; magnification: ×25. (A) Cell nucleus with intact DNA. (B) Cell nucleus with strongly degraded DNA. (C,D) Cell nucleus of A and B after software analysis; total DNA is encircled in red; green curve: head DNA intensity, blue curve: tail DNA intensity, yellow curve: total DNA intensity. (E) Mean relative tail DNA as percentage of total DNA per exposure time group and positive control (+H_2_O_2_); bars: standard deviation; ns: not significant; ***p < 0.001.

DNA damage measured as relative DNA in the tail as a percentage of DNA in the whole comet did not differ significantly between 2 or 24 h exposure and the respective sham control (2 h: *p* = 0.644; 24 h: *p* = 0.987; Figure 4E), whereas the H_2_O_2_‐treated positive controls differed significantly from sham controls (*p* < 0.0001; Figure 4E). Interestingly, DNA damage measured after 2 h incubation (exposure: 14 ± 2%; sham: 15 ± 4%; mean ± std. dev.) differed from that measured after 24 h incubation (exposure: 25 ± 9%; sham: 25 ± 6%), independent from exposure. This difference was slightly significant (exposure: *p* = 0.048; sham: *p* = 0.040).

### 3.3 Chromosomal Maldistribution

**Figure 5:**
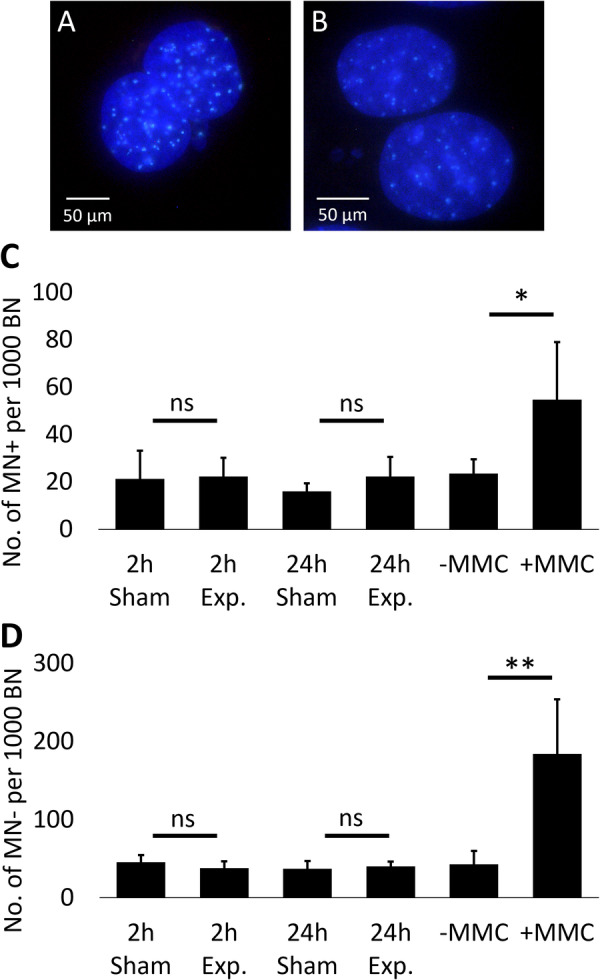
Results of micronucleus test for chromosomal aberrations. (A,B) Images of cell nuclei of binucleated cells with micronuclei stained with DAPI (blue) and anti‐kinetochore antibody (green); magnification: ×40 (oil immersion). (A) CREST‐positive micronuclei. (B) CREST‐negative micronuclei. (C,D) Mean number of binucleated cells with CREST‐positive (MN+; C) and CREST‐negative micronuclei (MN‐; D) per 1000 binucleated cells (BN) per exposure time group and positive control treated with mitomycin C ( + MMC); bars: standard deviation; ns: not significant; *p < 0.05; **p < 0.01.

The mean number of MN+ per 1000 BN did not differ between sham exposure samples and exposure samples for both time groups (2 h: *p* = 0.894; 24 h: *p* = 0.212; Figure 5C). The number of MN‐ per 1000 BN was approximately double as high as the number of MN+ and did also not differ between sham exposure samples and both exposure time groups (2 h: *p* = 0.278; 24 h: *p* = 0.599; Figure 5D). The samples of the positive control showed a highly increased number of MN+ and MN‐ per 1000 BN but also a high variation between replicates so that the difference between sham and exposure was only slightly significant for MN+ (*p* = 0.047), but highly significant for MN‐ (*p* = 0.008; Figure 5C,D).

## 4 Discussion

Our results indicate that cell viability was not affected by ELF‐MF at 50 Hz and 200 µT. This is in concordance with earlier findings in HaCaT cells, which were exposed to 50 Hz, 1 mT (Patruno et al. 2015; Vianale et al. 2008). One of the most recent in vitro studies about the effects of ELF‐MF indeed reports an increased cell viability after long‐term exposure (up to 96 h) of human B lymphoblastoid cells to 50 Hz, 10–500 µT in single experiments (Nguyen et al. 2023). However, these findings could not be confirmed by replication experiments within the same study. Another recently published study also found significant effects of ELF‐MF on cell viability of human breast cells exposed to 50 Hz, 0.1 or 1.0 mT when sampling occurred 96 h after starting the exposure with different exposure times (Elexpuru‐Zabaleta et al. 2023). These findings are relined by significant differences in mitochondrial membrane potential and reactive oxygen species. However, this study has various shortcomings including the lack of explanations about EMF shielding measures, the usage of electrical temperature loggers within the cell culture itself and its possible interaction with the exposure system. Furthermore, the experiments were not blinded, and no sham exposure was carried out; only a negative control. Nevertheless, it would be interesting to repeat these long‐term experiments with the blinded setting presented here.

We did not find any hint on differences in the relative amount of DNA strand breaks between exposed and sham‐exposed samples. This supports the results of earlier studies in human diploid fibroblasts exposed to 50 Hz, 1 mT intermittent for 24 h (Scarfí et al. 2005), human lens epithelial cells exposed to 50 Hz, 400 µT for up to 48 h (Zhu et al. 2016), and human neuroblastoma cells exposed to 50 Hz, 100 µT for 24 h (Mustafa et al. 2022). Although several study results in other cell types than keratinocytes also suggest that there is no effect, it has been pointed out that different cell types respond differently to the exposure (Simkó, Kriehuber, Weiss, et al. 1998). Therefore, one must be careful not to generalize these results or those obtained in other cell types.

The fact that DNA damage after 2 h exposure differed from that after 24 h exposure caught our interest. This observation was independent from exposure or sham exposure and must therefore have originated from side effects. When comparing the measurement data with previous publications on alkaline comet assay results in HaCaT cells (e.g., Hintzsche et al. 2012: 2%–10%; Sato and Sato 2022: 3%–5%), it is noticeable that the mean values of the relative tail DNA after 2 h in this study are relatively high (14%–15%). This could be due to the fact that the evaluation parameters differ slightly from each other but could also reflect a biological difference. The comet assay experiments were conducted blockwise, first 24 h, then 2 h experiments. Due to a contamination issue, an intensive incubator and lab cleaning and disinfection process was performed just before starting the comet assay 2 h experiments. Since all disinfection reagents could potentially be harmful to any kind of cell culture (be it an unwanted yeast culture or the cultured cell line itself), this could have led to the observed results. All disinfections were removed carefully after cleaning, and a fresh aliquot of cells was thawed; however, we might have missed residual toxic reagents or accidentally added an increased amount of disinfection reagent (Incuwater‐Clean, AppliChem, Darmstadt, Germany) to the incubator water bath. Apart from that, it is possible that the HaCaT cells in our study were, in general, already not in a very good overall condition at the beginning of the exposure/sham exposure, which continued over time, resulting in more comets occurring after 24 h than after 2 h. Maybe this effect could have been prevented by an additional cell culture medium change shortly before exposure, but as this would also discard positive factors released into the medium by the cells themselves, this has been deliberately omitted. It should be emphasized again that the differences mentioned are only differences between the exposure durations and not differences between the exposed samples and the corresponding sham controls. Following the above‐mentioned argumentation, this would mean that pre‐stressed cells were not influenced by the exposure.

Our results from the micronucleus test suggest that exposure to ELF‐MF does not lead to chromosomal maldistribution in keratinocytes. Although there are some study results on the effects of high‐frequency EMF on HaCaT cells and fibroblasts (e.g., Franchini et al. 2018; Hintzsche et al. 2012), there is only sparse data available for the effect of ELF‐MF on micronucleus formation. One example is the study of Udroiu et al. (2006) who stated that long‐term ELF exposure (50 Hz, 650 µT, 21 days) caused a two‐fold increase in CREST‐negative micronuclei and a four‐fold increase in CREST‐positive micronuclei in liver and peripheral blood samples from newborn mice whereas no such effect was found in exposed adults. Even though the study design and the tested cell types were different to ours, this study opens the idea of investigating cells from newborn individuals who might be more sensitive to external stressors than adult ones.

## 5 Conclusion

Taken together, our data on the effects of 50 Hz, 200 µT ELF‐MF exposure of HaCaT cells for up to 24 h supports the assessment that ELF‐MF do not cause health effects. Our study contributes valuable knowledge to the existing pool of evidence for the effects of ELF‐MF on human skin cells.

## Author Contributions

**Isabel Alexandra Gronau, Vivian Meyer**, and **Alexander Lerchl:** conceptualization. **Vivian Meyer:** data curation, formal analysis: **Isabel Alexandra Gronau**, **Vivian Meyer**, and **Karen Drees:** methodology. **Isabel Alexandra Gronau**, **Vivian Meyer**, and **Alexander Lerchl:** funding acquisition. **Isabel Alexandra Gronau**, **Vivian Meyer**, and **Karen Drees:** investigation. **Isabel Alexandra Gronau**, and **Vivian Meyer:** project administration. **Isabel Alexandra Gronau**, **Alexander Lerchl**, and **Vivian Meyer:** resources. **Alexander Lerchl:** supervision. **Vivian Meyer:** visualization. **Vivian Meyer:** writing – original draft. **All authors:** writing – review and editing.

## Conflicts of Interest

The authors declare no conflicts of interest.
